# Subjective cognitive complaint in at-risk mental states

**DOI:** 10.1016/j.scog.2025.100409

**Published:** 2025-12-03

**Authors:** Mirvat Hamdan-Dumont, Pénélope Chardac, Marine Habert, Anne-Laure Virevialle, Jérémy Couturas, Benjamin Calvet

**Affiliations:** a Centre Hospitalier Esquirol, Limoges, 87000, France; b Inserm UMR1094, IRD U270, Univ. Limoges, CHU Limoges, EpiMaCT- Epidemiology of chronic diseases in tropical zone, Institute of Epidemiology and Tropical Neurology, OmegaHealth, Limoges, 87000, France; c Université de Limoges, Faculté de médecine, 87000, Limoges, France

## Abstract

• There is poor literature concerning subjective cognitive complaint is ARMS
• Link is not clear between subjective and objectified cognitive issues
• We conducted a scoping review following PRISMA-ScR guidelines
• Two of three articles showed positive correlation between these two aspects.

Insight is a complex topic in schizophrenia. While the link between positive symptoms and their insight, the “cognitive insight”, has been developed (Dam, 2006; Van Camp et al., 2017), the connection between cognitive impairments and their awareness is weaker. Insight has several facets; we will call the awareness of cognitive symptoms the subjective cognitive complaint (SCC) (Johnson et al., 2009). Around 50 % of schizophrenia subjects are aware of their cognitive symptoms (Medalia and Thysen, 2008). At-risk mental states (ARMS) represent a group of subjects experiencing psychotic symptoms attenuated in frequency and intensity (Drake and Lewis, 2005). The risk of transition from ARMS to a psychotic episode is around 20 % (95 % CI 17–25 %) at 2 years (Fusar-Poli et al., 2017). However, cognitive and functional alterations are often already present (de Paula et al., 2015; Simon et al., 2012) but the literature to date remains poor regarding the link between SCC and measured cognitive disturbances, as well as the impact within real life functioning. Yet, in this population, these complaints are particularly useful as they may represent early warning signals of neurocognitive disorders associated with emerging psychosis (Fusar-Poli et al., 2013). ARMS individuals frequently complain of difficulties in areas such as memory, attention and executive functions, which may contribute to overall functional deterioration (Lencz et al., 2003). These cognitive complaints are often correlated with an increase in subclinical psychotic symptoms (Bolt et al., 2019). Furthermore, SCC must be distinguished from the concept of basic symptoms, a more global and phenomenological approach of the prodromal stages (Huber and Gross, 1989). A few questionnaires for assessing SCC are currently in use in schizophrenia, such as Self-Assessment Scale of Cognitive Complaints in Schizophrenia SASCCS (Johnson et al., 2009), Schizophrenia Cognition Rating Scale SCoRS (Keefe et al., 2006), the Measure of Insight into Cognition-Self Report MIC-SR (Saperstein et al., 2012) and Subjective Scale To Investigate Cognition in Schizophrenia SSTICS (Cella et al., 2020). We therefore searched the literature for studies examining SCC in ARMS and investigating a potential link between SCC and measurable cognitive impairment.

The following electronic databases were searched: Pubmed, Science Direct, Scopus and Google Scholar. The following term search was demanded in Pubmed: ((“ultra-high risk” OR “clinical high risk” OR “prodromal psychosis” OR “at-risk mental states” OR “basic symptoms” OR “psychosis risk syndrome”) AND (“insight” OR “unawareness”) AND (“cognitive impairment” OR “cognitive issues”)). The combination of terms has been slightly modified for Science Direct, Scopus and Google Scholar to fit the maximal characters allowed: ((“ultra-high risk” OR “clinical high risk” OR “prodromal psychosis” OR “at-risk mental states” OR “basic symptoms”) AND (“insight” OR “unawareness”) AND (“cognitive impairment” OR “cognitive issues”)). No restrictions on publication date or language were applied, nor were any restrictions on age or gender of participants. Titles and abstracts were screened by two independent reviewers for the first selection, and full texts were considered in the second step by each one of them. In case of doubt, a third opinion was sought. For each publication, following data have been extracted: name of the study, sample size, ARMS assessment method, age of the participants, gender ratio, SCC assessment method, OMC (Objectively Measured Cognition) tool, results and association between SCC and OMC. Data collection, extraction and reporting followed the Preferred Reporting Items for Systematic Reviews and Meta-Analyses extension for Scoping Reviews guidelines (PRISMA-ScR) (Tricco et al., 2018). Study quality was assessed using the Standard Quality Assessment tool QualSyst tool for quantitative studies (Kmet LC and Lee, 2004).

The initial search provided 102, 342 records, from which 121 duplicates were removed. Were then excluded 102, 209 references on title and abstract, leaving 12 full-text articles assessed for eligibility. Reports were excluded, if neurocognition was compared to cognitive insight (*n* = 5), or social cognition (*n* = 2) in ARMS. One of the excluded articles dealt with phenomenology, while another dealt with auditory processing. We thus selected 3 papers: Higuchi et al. (Higuchi et al., 2017), Glenthøj et al. (Glenthøj et al., 2020) and Randers et al. (Randers et al., 2020). All studies included ARMS participants assessed with the CAARMS (Yung et al., 2005; Miyakoshi et al., 2009). SCoRS was used to assess SCC in two papers, whereas MIC-SR was the assessment tool in the third. The age of the participants was spread between 18 and 24. In 2 of the selected studies, SCC was compared with a composite score of objectifiable performance using the Brief Assessment of Cognition in Schizophrenia (BACS) (Kaneda et al., 2007; Keefe et al., 2004), which enabled to assess various neurocognitive domains from a global perspective, namely verbal memory and fluency, working memory, motor functions, selective and sustained attention, and executive functions. Randers et al. used a composite score on the Behavior Rating Inventory of Everyday Functioning- Adult Version (BRIEF-A) (Roth et al., 2014) as well as a composite score on a battery of cognitive tests and chose to compare the SCC with the objective assessment of executive functions, important for everyday functioning. Two of the 3 studies, Higuchi et al. and Randers et al., showed that there was a direct correlation between SCC and objective impairment of cognitive function. Glenthøj et al. found no significative correlation between MIC-SR score and the composite score. Randers et al. also showed significative correlation between SCoRS and executive functioning. The scores obtained using the QualSyst tool were 0.64 for Randers et al., Higuchi et al., and 0.54 for Glenthøj et al. All the information collected are available in Table 1.

**Table 1:** Characteristics of the studies included in the scoping review.

Article, year of publication, country & ualSyst score	Sample size (N)	ARMS Diagnosis tool	Age	Sex ratio	SCC Tool	OMC tool	Results
Glenthøj et al., 2020 (DK) 0.54	52	CAARMS	24.0 (4.6)	29F/23M	MIC-SR	BACS	- MIC-SR total = 21.2(7.6) - BACS composite score = −1.04(1.17) - 32 subjects showed impaired measured cognition z-score ≤ 1.35 - No correlation between MIC-SR and the BACS composite score *r* = −0.12 *p* = 0.39
Higuchi et al., 2017(JP) 0.64	102	CAARMS	19.4 (3.9)	38F/64M	SCoRS	BACS	- SCoRS total = 3.7(1.9) - BACS composite score = −0.48(0.94) - Correlation between SCORS and BACS composite z-score *r* = 0.276 *p* = 0.005
Randers et al., 2020(DK) 0.64	39	CAARMS	23.4 (4.4)	22F/17M	SCoRS	Comprehensive cognitive test battery and BRIEF-A	- 25 subjects confronted to SCoRS total = 4.4(1.7) - 39 subjects had a cognitive composite z-score = −1.02(1.11) - 22 subjects confronted to BRIEF-A = 143.5(22.2) - Correlation between SCoRS and cognitive composite z-score r = −0.343 *p* = 0.037 - Correlation between SCoRS and executive function z-score *r* = −0.343 *p* = 0.033

Abbreviations: DK Denmark, JP Japan, OMC objectively measured cognition, SCC subjective cognitive complaint, CAARMS Comprehensive Assessment of At-risk Mental State, MIC-SR Measure of Insight into Cognition-Self Report, SCORS Schizophrenia Cognition Rating Scale, BACS Brief Assessment of Cognition in Schizophrenia, BRIEF- A Behavior Rating Inventory of Everyday Functioning – Adult Version.

These results highlight a possible link between SCC and objective measures of cognitive impairment in ARMS patients, as two of the 3 selected studies found a correlation between SCC and measured cognitive impairment. This link has already been explored in full-blown schizophrenia, although the results are not conclusive (Medalia and Thysen, 2008; Kaneda et al., 2011), possibly due to a loss of awareness as the disease progresses (Xu et al., 2022). Nevertheless, individuals suffering from ARMS do not tackle their problems in the same manner as those diagnosed with schizophrenia. A link between cognitive impairment and the risk of transition has already been demonstrated (Catalan et al., 2021). Thus, if subjects with ARMS are aware of their cognitive disorders and if the SCC is reliable, it would be possible to easily identify patients at increased risk of transition in current clinical practice using rapid subjective questionnaires such as the SCoRS.

Our review has several limitations. QualSyst scores obtained are considered adequate, but do not reach the cut-off of 0.75 suggested by the authors for a review (Kmet LC and Lee, 2004). The sample sizes are small, though typical of studies concerning ARMS, given the difficulties of detection and the selection bias of help-seeking in this population. None of these studies had determined the required sample size in advance, making it difficult to draw conclusions about the statistical power of the results. However, since neuropsychological test batteries are usually carried out by trained neuropsychologists, psychologists, or assistants — and, in some countries, by trained psychiatrists — and since these tests are time-consuming and require the subject to be relatively symptom-stabilized, incorporating a quicker subjective test, such as the SCoRS, into clinical practice could be more feasible, particularly for early intervention services. In addition, these rapid questionnaires can easily be incorporated into clinical studies and could provide an interesting parameter for monitoring ARMS evolution over time. There would therefore be a direct interest in translating and validating these scales in several languages, in both high-income and low-income countries.

## CRediT authorship contribution statement

**Mirvat Hamdan-Dumont:** Writing – original draft, Methodology, Conceptualization. **Pénélope Chardac:** Writing – original draft. **Marine Habert:** Writing – review & editing. **Anne-Laure Virevialle:** Writing – review & editing. **Jérémy Couturas:** Writing – review & editing. **Benjamin Calvet:** Writing – review & editing, Validation, Methodology.

## Funding

This research did not receive any specific grant from funding agencies in the public, commercial, or not-for-profit sector.

## Declaration of competing interest

The authors declare that they have no known competing financial interests or personal relationships that could have appeared to influence the work reported in this paper.
